# Effect of the Microstructure of Carbon Supports on the Oxygen Reduction Properties of the Loaded Non-Noble Metal Catalysts

**DOI:** 10.3390/nano15171327

**Published:** 2025-08-29

**Authors:** Dan Ma, Yudong Zhang, Menghan Liang, Runyu Niu, Yao Ge, Yanan Zou, Xiaorui Dong

**Affiliations:** 1 School of Energy and Power Engineering, North University of China, Taiyuan 030051, China; sz202416039@st.nuc.edu.cn (D.M.); sz202216007@st.nuc.edu.cn (M.L.); sz202316004@st.nuc.edu.cn (R.N.); 2 Chongqing University Industrial Technology Research Institute, Chongqing 400030, China; geyao@cqu.edu.cn; 3 College of Mechanical Engineering, University of South China, Hengyang 421001, China; zouyn@usc.edu.cn

**Keywords:** non-noble metal catalysts, carbon supports, pyrolysis temperature, microstructure, oxygen reduction reaction

## Abstract

The development of efficient non-noble metal catalysts is critical for advancing sustainable fuel-cell technologies. This study investigates the effect of carbon support microstructure on the oxygen reduction reaction (ORR) performance of Fe-N-C catalysts. By precisely tuning the pyrolysis temperature of activated carbon (AC) between 600 and 1000 °C, we elucidate the mechanistic influence of the physicochemical characteristics of the carbon support on the ORR activity of the supported catalyst. Increasing the pyrolysis temperature enhanced the electrical conductivity of the carbon support, thereby improving the ORR performance of the catalyst. However, while the defect density and specific surface area of the carbon support initially increased with increasing pyrolysis temperature, they declined when elevated temperatures were used (e.g., 1000 °C), leading to reduced ORR activity. The AC-900 support, pyrolyzed at 900 °C, exhibited an optimal balance of a high surface area, abundant defects, and superior conductivity. An Fe phthalocyanine/AC-900 catalyst synthesized using the AC-900 support exhibited excellent ORR activity (*E*_1/2_: 0.89 V and *E*_on_: 0.95 V vs. reversible hydrogen electrode (RHE)) in 0.1 M KOH. This work highlights the pivotal role of carbon support microstructure in governing the ORR activity of the supported catalyst and provides a rational strategy for designing high-performance non-noble metal electrocatalysts.

## 1. Introduction

Fuel-cell technology has emerged as a promising sustainable energy solution to address growing global energy demand and environmental challenges [1,2]. While platinum–carbon (Pt/C) catalysts remain the benchmark for catalyzing the oxygen reduction reaction (ORR) in fuel-cell cathodes [3,4], their practical application is severely restricted by their high cost, susceptibility to poisoning, and the limited natural abundance of Pt [5,6]. These limitations have stimulated intensive research efforts to develop cost-effective, durable, and high-performance non-precious metal ORR catalysts [7,8]. Among the various alternatives, M-N-C catalysts (where M represents a transition metal) have shown particular promise, demonstrating ORR activities comparable to those of Pt/C while offering additional advantages, including natural abundance, lower cost, and superior resistance to poisoning [9,10].

Loading ORR-active M-N structures onto porous carbon matrices represents an effective and scalable strategy for the synthesis of M-N-C catalysts [11]. As catalyst supports, porous carbon materials critically determine the physicochemical properties of the resulting M-N-C catalysts, including their specific surface area, pore structure, and electrical conductivity [12]. Zhang et al. [13] pioneered this approach by developing a lamellar porous carbon support for loading iron phthalocyanine (FePc). The resulting FePc/PBC catalyst maintained the parent carbon’s high surface area and 3D interconnected porous architecture, enabling the full utilization of FePc active sites and thus an exceptional ORR performance (*E*_1/2_ = 0.91 V vs. RHE), limiting current density J_L_ = 5.03 mA cm^−2^). In a complementary study, Chai et al. [14] employed highly conductive carbon nanotubes (CNTs) as a support to fabricate Fe0.1-CNT@NHC catalysts containing Fe_7_C_3_ nanoparticles. The superior conductivity of the CNT framework facilitated efficient charge transport, resulting in an outstanding J_L_ of 6.08 mA cm^−2^ in rotating disk electrode (RDE) measurements. These studies collectively demonstrate that the structural and electronic properties of carbon supports fundamentally govern the ORR activity of non-noble metal catalysts.

The selection of porous carbon supports plays a critical role in determining the ORR performance of non-noble metal catalysts because of the widely varying microstructures and physicochemical properties of carbon structures. Studies have demonstrated that different carbon substrates lead to significant differences in the catalytic activity of a non-noble metal catalyst. For instance, Cui et al. [15] synthesized a non-noble metal catalyst by depositing cobalt on a porous tubular carbon support. The well-developed pore structure of the resulting catalyst facilitated efficient mass transport pathways for O_2_ diffusion and electrolyte permeation, resulting in a remarkable ORR onset potential (*E*_on_) of 0.96 V and an *E*_1/2_ of 0.85 V vs. RHE. Furthermore, Meng et al. [16] developed an Fe-Nx@NC/reduced graphene oxide catalyst by electrospinning a highly conductive porous carbon support loaded with FeCl_3_. The superior electrical conductivity of the carbon substrate contributed to an outstanding ORR activity, as evidenced by an *E*_1/2_ of 0.925 V vs. RHE in RDE tests. Thus, the findings of earlier studies highlight that variations in the microstructural and physicochemical properties of porous carbon supports significantly influence the ORR performance of the non-metal catalysts loaded on them. While previous studies have partially elucidated the effects of the pore size and specific surface area, a comprehensive understanding of the effects of other key properties—such as oxygen-containing functional groups, electrical conductivity, and pore-size distribution—on the ORR activity of carbon-supported non-noble catalysts is lacking. Therefore, it is important to systematically investigate these factors to identify the optimal characteristics of a carbon support for designing high-performance non-noble metal ORR catalysts, addressing a critical scientific challenge in this field.

In recent years, non-noble metal catalysts have made significant progress in the field of ORR in anion exchange membrane fuel cells (AEMFCs). Research shows that the conductivity of carbon-based materials and the oxygen-containing functional groups on their surfaces have significant influences on ORR activity. For instance, Kostuch et al. found that by regulating the carbonization temperature of the carbon carrier (650–1050 °C), the synergistic effect of conductivity and surface carbonyl/quinone functional groups determined the ORR performance of the catalyst. Among them, the samples carbonized at 850 °C exhibited the best volcanic activity [17]. Furthermore, Ibrahim et al. developed a Fe-N-C catalyst prepared by an ionic thermal synthesis method. Its mesoporous structure and high-density Fe-Nx active sites enable it to exhibit excellent ORR activity in alkaline media, with a *E*_1_/_2_ of 0.90 V vs. RHE, and a peak power density of 599 mW cm^−2^ was achieved in the single-cell test [18]. These studies have emphasized the significance of optimizing the structure of carbon carriers and the design of active sites, providing new ideas for the development of low-cost and high-performance AEMFC cathode catalysts. Sanetuntikul and Shanmugam synthesized Fe-N-C and Co-N-C catalysts by high-pressure pyrolysis. Their specific surface areas were 377.5 and 369.3 m^2^ g^−1^, respectively, and they had rich mesoporous structures. XPS analysis indicated that there were N-C and M-N <s:1> active sites in the material. Among them, the *E*_1/2_ of Fe-N-C (0.86 V was even superior to that of commercial Pt/C catalysts (0.84 V), and it only decayed by 4 mV after 5000 cycles, demonstrating excellent stability. Furthermore, the rotating ring-disk electrode (RRDE) test confirmed that its ORR process mainly proceeds through a four-electron path, with an extremely low H_2_O_2_ yield (<3%). In the single-cell test, the maximum power density of Fe-N-C (75 mW cm^−2^) was close to Pt/C (80 mW cm^−2^), further verifying its practical application potential [19]. As carbon supports are typically synthesized by carbonizing organic precursors, the pyrolysis temperature critically governs their microstructural and physicochemical properties. In this study, we systematically modulated the key attributes of carbon materials—including their specific surface area, porosity, pore-size distribution, and electrical conductivity—by controlling the pyrolysis temperature. Our objective was to elucidate the structure–activity relationship between the microstructure of the carbon support and the ORR performance of the Fe-N-C catalyst, with a particular focus on the underlying mechanisms through which these properties influence the catalytic behavior.

This work is not aimed at preparing a high-performance ORR catalyst, but rather at establishing a clear correlation between the microstructure and physicochemical properties of the carbon support and the catalytic effect of the non-metallic catalyst it supports. The findings provide fundamental insights into the rational design of M-N-C catalysts, offering a theoretical framework for optimizing their synthesis and performance. This study reveals that the nonlinear relationship among pyrolysis temperature, carbon structure, and catalytic activity, demonstrating that 900 °C represents a performance turning point rather than a simple positive correlation. Specifically, AC-900 simultaneously achieves a high specific surface area (1031.8 m^2^/g), a high defect density (I_D_/I_G_ = 0.95), and high electrical conductivity (2.37 × 10^4^ S/m). These results confirm that the synergistic optimization of these three factors, rather than any single parameter, is the key to enhancing ORR activity.

## 2. Materials and Methods

### 2.1. Preparation of Carbon Precursors

First, 1 g of activated carbon (AC, Aladdin, CAS number 7440-44-0, Shanghai, China) was weighed and placed in a ceramic boat, which was then covered and loaded into a tube furnace. The sample was heated to 600, 700, 800, 900, or 1000 °C at a heating rate of 5 °C min^−1^ under a N_2_ flow rate of 200 sccm and held there for 2 h. Thereafter, the sample was cooled to room temperature, and the product was collected. The samples pyrolyzed at 600, 700, 800, 900, and 1000 °C are denoted as AC-600, AC-700, AC-800, AC-900, and AC-1000, respectively.

### 2.2. Preparation of Catalysts

A total of 100 mg AC was subjected to solvothermal treatment in 50 mL of a 0.5 mg mL^−1^ FePc (MACKLIN, CAS number 132-16-1, Shanghai, China) solution in anhydrous ethanol at 180 °C for 12 h. Then, the sample was cooled to room temperature, repeatedly washed with anhydrous ethanol and deionized water to remove soluble impurities, and dried at 60 °C for 6 h. Finally, the dried sample was ground into a powder. The resulting samples are denoted as FePc/AC-600, FePc/AC-700, FePc/AC-800, FePc/AC-900, and FePc/AC-1000, according to the pyrolysis temperature of AC. Solvothermal treatment and heat treatment were performed using a solvothermal reactor (DZF-6020AB, Shandong Ruyi Scientific Instrument Co., Ltd., Jinan, China) and a high-temperature tube furnace (BTF-1200C, BEQ Company, Hefei, China), respectively, with temperature control accuracies of ±0.1 °C and ±1 °C.

### 2.3. Physical and Chemical Property Analysis

The microstructures of AC-900 and FePc/AC-900 were examined using field-emission scanning electron microscopy (SEM). The specific surface area and pore-size distribution of the AC samples prepared at different carbonization temperatures were determined by N_2_ physisorption measurements using a fully automated Micromeritics ASAP 2460D analyzer. The Brunauer–Emmett–Teller method was used to calculate the specific surface area from the N_2_ adsorption–desorption isotherms, while pore-size distributions were derived using the Barrett–Joyner–Halenda model.

Fourier transform infrared (FTIR) spectroscopy (Nicolet IS10) was employed to analyze the functional groups present in the AC to AC-1000 series. The specific steps are as follows: pre-dried KBr was ground into powder and pressed into a blank tablet (15 MPa, 20 s), for background spectrum collection. Catalyst samples (~1 wt.%) were homogenized with KBr, then processed identically into pellets for analysis. All molds were cleaned with anhydrous ethanol to prevent cross-contamination. Consistent instrumental parameters were maintained throughout testing to ensure data reliability. The crystalline structures of FePc and FePc/AC-600 to FePc/AC-1000 were characterized by X-ray diffraction (XRD; Bruker D8 Advance). X-ray photoelectron spectroscopy (XPS; Thermo Scientific Escalab 250Xi) was conducted to determine the elemental composition and functional group contents of the FePc/AC-600 to FePc/AC-1000 catalysts. Raman spectroscopy (Horiba LabRAM HR Evolution) was used to evaluate the graphitization degree and defect density of the materials. Additionally, the electrical conductivities of the AC-600 to AC-1000 samples were measured using a standard four-point probe method.

### 2.4. Evaluation of the Electrochemical Performance

The ORR activities of the catalysts were evaluated using a Bio-Logic VMP3 electrochemical workstation (France) with a RRDE system (Pine Research Instruments, Durham, NC, USA). Measurements were conducted in a 0.1 M KOH electrolyte using a standard three-electrode configuration, where the catalyst-coated RRDE (5 mm diameter glassy carbon disk with a Pt ring) served as the working electrode (WE), Hg/HgO electrode as the reference electrode (RE), and a graphite rod served as the counter electrode. All measured potentials were converted to the RHE scale using the following relationship:

*E*_RHE_ = *E*_Hg/HgO_ + 0.098 V + 0.0591 × pH (1)

A total of 10 mg of the catalyst was weighed into a sample vial, followed by the addition of 100 μL of 5% Nafion solution and 2.9 mL of a 1:1 (*v*/*v*) mixture of deionized water and isopropyl alcohol. The resulting slurry was ultrasonicated for 60 min to achieve uniform dispersion. For catalyst loading on the glassy carbon electrode, 20 μL of the prepared slurry was pipetted onto the pre-treated glassy carbon electrode surface and dried at 25 °C for 120 min. The resulting electrode loading was 0.27 mg/cm^2^.

Before each measurement, the working electrode was subjected to 30 cycles of cyclic voltammetry (CV) between 0.05 and 1.2 V vs. RHE at 50 mV s^−1^ in a N_2_-saturated electrolyte to achieve a stable electrochemical surface. The WE position was carefully maintained at a constant height to ensure consistent solution resistance between the RE and the WE.

ORR polarization curves were obtained through linear sweep voltammetry (LSV) measurements conducted at 1600 rpm at a scan rate of 10 mV s^−1^ in the potential range of 0.05 to 1.2 V vs. RHE. Background currents were first recorded in a N_2_-saturated electrolyte, following which ORR measurements were conducted in an O_2_-saturated electrolyte. The Faradaic ORR current was determined by subtracting the background current from the measured ORR current.

The electron transfer number (n) and hydrogen peroxide yield (%H_2_O_2_) were calculated from the simultaneous disk and ring currents, using the following equations [20,21]:

n = (4*i*_Disk_)/(*i*_Disk_ + *i*_Ring_/N) (2)

%H_2_O_2_ = 100 × (2 *i*_Ring_/N)/(*i*_Disk_ + *i*_Ring_/N) (3)

where *i*_Disk_ is the disk current, *i*_Ring_ is the ring current, and N is the collection efficiency of the RRDE apparatus.

## 3. Results and Discussion

### 3.1. Physical and Chemical Properties of the Carbon Precursors and Catalysts

**Figure 1:**
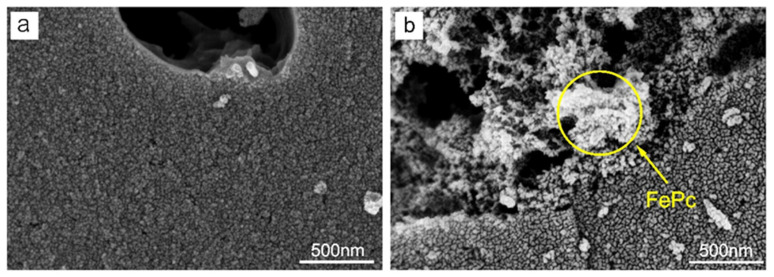
SEM images of (**a**) AC-900 and (**b**) FePc/AC-900.

Figure 1 presents the morphological characteristics of AC-900 and FePc/AC-900. The pristine carbon material (AC-900, Figure 1a) displays micrometer-sized carbon particles with smooth surfaces. In contrast, FePc/AC-900 (Figure 1b) shows significant morphological differences, with particles agglomerated into block-like structures. This difference is due to the fact that during the 180 °C solvent heat treatment process, FePc molecules promote particle agglomeration through interfacial interactions [22]. The consequent loss of these functional groups enhances interparticle bonding, leading to carbon agglomeration and densification.

The formed agglomerates create irregular pore structures and defects between carbon particles (Figure 1b), which are particularly beneficial for catalytic applications. These structural features provide abundant catalytically active sites for electrochemical reactions, potentially enhancing the catalyst’s ORR activity [23]. Notably, the SEM micrograph reveals brighter domains (highlighted by yellow circles) that are attributed to FePc-enriched regions. This assignment is based on SEM contrast mechanisms, where brighter areas correspond to materials with lower electrical conductivity [24]. Given the distinct conductivity disparity between FePc (poor conductor) and the carbon support (good conductor)—the only two components in the catalyst—the morphological and contrast differences observed in the yellow-circled regions (relative to the carbon matrix) further support their identification as FePc-enriched domains.

**Figure 2:**
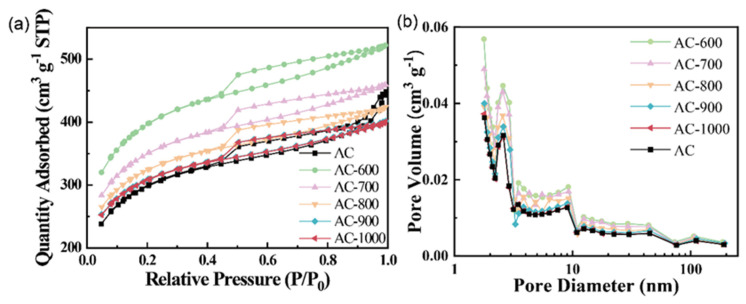
(**a**) N_2_ adsorption–desorption isotherms and (**b**) pore-size distribution for AC, AC-600, AC-700, AC-800, AC-900, and AC-1000.

Figure 2 presents the N_2_ adsorption–desorption isotherms of the carbon materials pyrolyzed at different temperatures and the corresponding pore-size distributions. All samples exhibit Type IV isotherms (Figure 2a), typical of mesoporous carbon materials [25], with pronounced hysteresis loops in the relative pressure (*P*/*P*_0_) range of 0.4 to 1.0, confirming the coexistence of mesopores [26]. In addition, the BET specific surface areas of AC, AC-600, AC-700, AC-800, AC-900, and AC-1000 were determined to be 1040.9, 1340.2, 1179.5, 1086.6, 1031.8, and 1030.5 m^2^ g^−1^, respectively. This trend of initial increase followed by a decrease in the BET specific area originates from competing thermal processes: (1) at 600 °C, the decomposition of oxygen-containing functional groups generates CO and CO_2_, creating additional pores and maximizing the surface area of the material [27]; (2) above 600 °C, structural collapse occurs, which partially blocks the pores, causing a gradual reduction in the surface area [28]. These findings are particularly relevant for ORR catalysis, as larger surface areas typically accommodate more active sites [29].

The BJH pore-size distributions (Figure 2b) demonstrate the predominance of 2–10 nm pores, along with the presence of both micropores (<2 nm) and mesopores (2–50 nm). This hierarchical porosity offers dual advantages: the micropores contribute substantially to surface area, while the mesopores promote mass transport by facilitating enhanced ion diffusion and electrolyte accessibility [30,31].

The electrical conductivities of the thermally treated carbon materials were systematically evaluated using the four-point probe method, and the results are summarized in Table 1. The measurements revealed a strong dependence of conductivity on the pyrolysis temperature. As the pyrolysis temperature increased, the conductivity increased by four orders of magnitude from 1.36 × 10^−3^ S/m for AC to 3.28 × 10^4^ S/m for AC-1000 under 2 MPa compression. The electrical conductivity under 2 MPa compression increased progressively across the series, as follows: AC (1.36 × 10^−3^ S/m) < AC-600 (2.62×10^−2^ S/m) < AC-700 (3.47 × 10^2^ S/m) < AC-800 (8.23 × 10^3^ S/m) < AC-900 (2.37 × 10^4^ S/m) < AC-1000 (3.28 × 10^4^ S/m).

The observed dramatic enhancement in conductivity originates from improved graphitic ordering at elevated temperatures, which facilitates electron transport through the carbon matrix [32].

**Figure 3:**
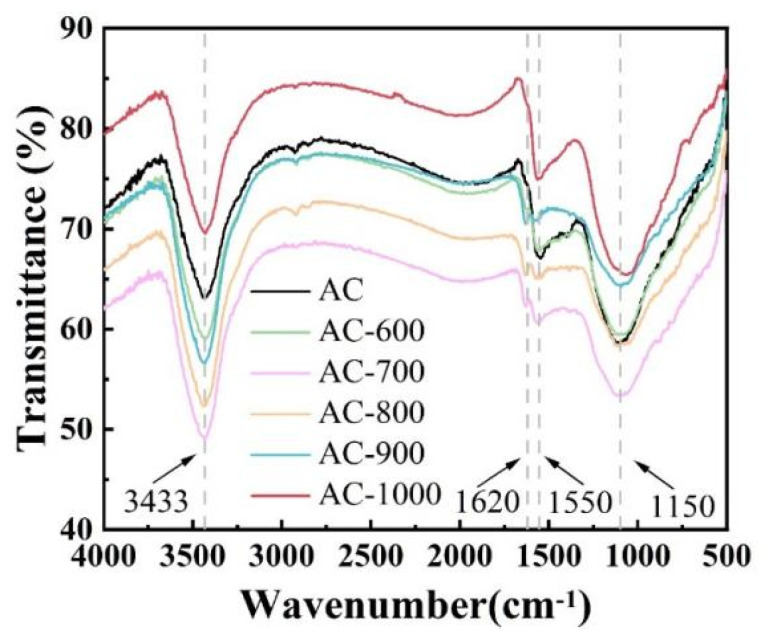
FT-IR spectra of AC, AC-600, AC-700, AC-800, AC-900, and AC-1000.

Figure 3 presents the FTIR spectra of the AC samples pyrolyzed at different temperatures. All samples display three characteristic absorption bands at 1100, 1550, and 1620 cm^−1^. The band at 1550 cm^−1^ corresponds to the fundamental carbon skeletal vibration (C-C) of the activated carbon matrix [33], while the peaks at 1150 cm^−1^ (C-O-C asymmetric stretching) and 1620 cm^−1^ (C=O stretching) are due to oxygen-containing functional groups [34]. The specific steps are as follows: pre-dried KBr was ground into powder and pressed into a blank tablet (15 MPa, 20 s) for background spectrum collection. Catalyst samples (~1 wt.%) were homogenized with KBr, then processed identically into pellets for analysis. All molds were cleaned with anhydrous ethanol to prevent cross-contamination. Consistent instrumental parameters were maintained throughout testing to ensure data reliability. The relative FTIR peak intensity analysis revealed the following trend in the oxygen functional group content: AC-700 > AC-800 > AC-600 > AC > AC-900 > AC-1000. This thermal treatment temperature-dependent behavior has significant implications in catalyst preparation. As oxygen functionalities serve as anchoring sites for FePc through π-π interactions [35], the FePc binding capacity of the carbon support follows the same order as its oxygen content. However, we note an important trade-off: while a higher oxygen content enhances metal coordination, excessive surface functionalization introduces structural defects that compromise electrical conductivity [36]. This is because increased oxygen content leads to a higher abundance of oxygen-containing groups (e.g., hydroxyl and carboxyl groups), which typically exhibit low electrical conductivity [37]. Remarkably, AC-900 demonstrates an optimal balance between these competing factors, maintaining sufficient oxygen groups for binding FePc while minimizing conductivity-limiting defects. This characteristic makes AC-900 particularly promising for ORR applications requiring both high catalytic activity and efficient charge transfer. Additionally, all spectra exhibit a broad absorption band at 3433 cm^−1^, corresponding to O-H stretching vibrations [38]. This feature indicates the presence of adsorbed water molecules, a common feature of carbon materials with well-developed porosity.

**Figure 4:**
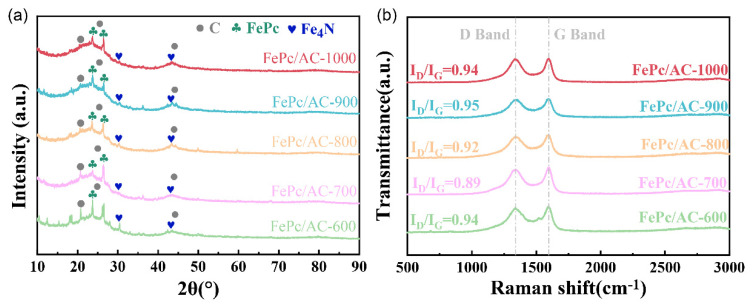
(**a**) XRD patterns and (**b**) Raman spectra of FePc/AC-600, FePc/AC-700, FePc/AC-800, FePc/AC-900, and FePc/AC-1000.

Figure 4a presents the XRD patterns of the FePc/AC catalysts synthesized using AC samples pyrolyzed at different temperatures (600–1000 °C). All samples exhibit three characteristic diffraction features: (i) two broad peaks centered at ~25° and ~43.7°, corresponding to the (002) and (100) planes of graphitic carbon [39], and (ii) a sharp peak at ~20.7°, attributed to the (111) plane. Additional peaks observed at ~30.2° and ~42.5° could be indexed to the (400) and (111) planes of Fe_4_N [40], while those at ~23.5° and ~26.5° confirm the successful incorporation of FePc [41]. The gradual decrease in the peak intensity of FePc with increasing carbonization temperature (FePc/AC-600 > FePc/AC-700 > FePc/AC-800 > FePc/AC-900 > FePc/AC-1000) suggests that thermally induced structural densification limits the accessibility of internal pores to FePc.

Complementary Raman spectral analysis (Figure 4b) revealed two characteristic bands at ~1350 cm^−1^ (D band, disorder-induced mode) and ~1580 cm^−1^ (G band, graphitic E_2_g mode) [42]. The intensity ratio of the D to G band (*I*_D_/*I*_G_) quantitatively reflects the structural order, and FePc/AC-900 exhibited the highest *I*_D_/*I*_G_ ratio (0.95) among the samples (FePc/AC-600: 0.94; FePc/AC-700: 0.89; FePc/AC-800: 0.92; and FePc/AC-1000: 0.94). This trend indicates that FePc/AC-900 has the highest defect density among the samples, which can result in enhanced ORR activity according to established structure–activity relationships [43,44,45]. The optimal defect concentration in FePc/AC-900 can contribute to a superior catalytic performance by providing abundant active sites, while the preserved graphitic domains can provide sufficient electrical conductivity.

However, a critical trade-off was observed; while higher conductivity promotes the formation of electrochemically active three-phase boundaries, excessive graphitization reduces defect concentration, as evidenced by Raman spectroscopy. Remarkably, AC-900 demonstrated an optimal balance between these two factors, achieving a high conductivity (2.37 × 10^4^ S/m) as well as substantial defect density (*I*_D_/*I*_G_ = 0.95). This unique combination of conductivity and defect density explains its superior ORR performance compared to that of the other samples, as discussed later in Section 3.2, because defect sites serve as catalytic centers while adequate conductivity ensures efficient charge transfer during the electrochemical reaction [46].

**Figure 5:**
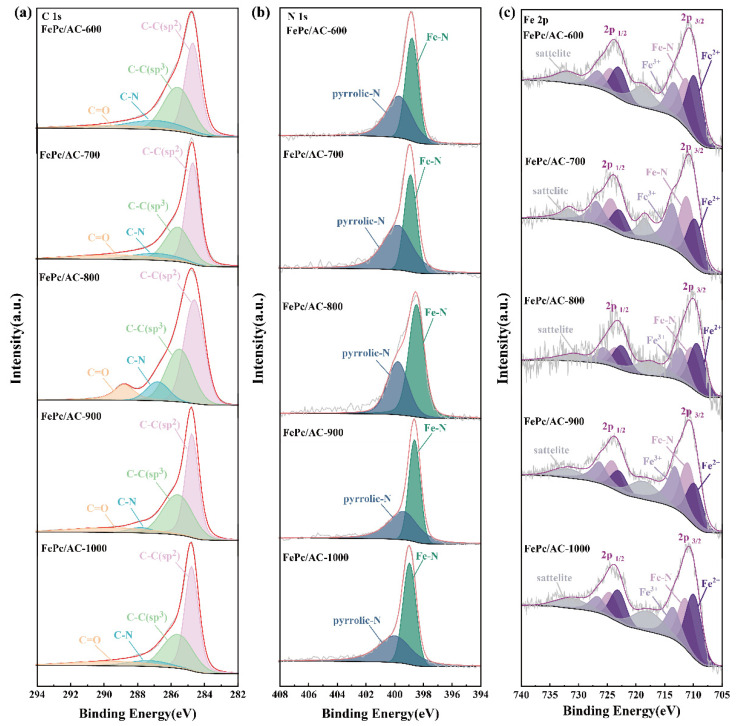
(**a**) C 1s, (**b**) N 1s, and (**c**) Fe 2p XPS profiles of FePc/AC-600, FePc/AC-700, FePc/AC-800, FePc/AC-900, and FePc/AC-1000.

Comprehensive XPS characterization was performed on the FePc/AC series (i.e., FePc/AC-600 to FePc/AC-1000) to elucidate the chemical compositions and bonding configurations of the samples. The deconvoluted C 1s spectra (Figure 5a) reveal the presence of four distinct carbon components: graphitic C-C (sp^2^, 284.8 eV), disordered C-C (sp^3^, 285.6 eV), C-N (286.9 eV), and C=O (289.3 eV) [47]. The predominance of sp^2^-hybridized carbon in all samples confirms the formation of conductive graphitic networks, which facilitate electron transfer during ORR processes [48].

The N 1s spectra (Figure 5b) reveal the presence of two characteristic nitrogen species: Fe-coordinated nitrogen (Fe-N, 399.2 eV) and pyrrolic nitrogen (400.2 eV) [49]. Quantitative analysis revealed the following Fe-N content distribution:

FePc/AC-900 (55.94%) > FePc/AC-1000 (54.98%) > FePc/AC-600 (53.10%) > FePc/AC-800 (48.80%) > FePc/AC-700 (44.40%)

The high-resolution Fe 2p spectra (Figure 5c) exhibit three characteristic doublets corresponding to Fe^3+^ (714.1/727.3 eV), Fe-N coordination (711.2/724.4 eV), and Fe^2+^ (709.8/723.0 eV), along with the associated satellite peaks (718.9/732.1 eV) [50]. FePc/AC-900 contained the highest Fe-N coordination content (30.8%), significantly surpassing those of the other samples (FePc/AC-600: 27.9%; FePc/AC-700: 29.5%; FePc/AC-800: 25.8%; and FePc/AC-1000: 25.9%). Notably, the XPS measurement process inherently involves a certain degree of error. In the present study, the 5% difference in Fe-N content between FePc/AC-900 (30.8%) and FePc/AC-800 (25.8%) falls well within this acceptable error range. This finding is particularly significant as Fe-N moieties are well-established as the primary active sites for ORR, with their concentration being directly proportional to catalytic activity [51]. The combined spectroscopic results conclusively suggest that FePc/AC-900 exhibits superior ORR activity owing to its optimal Fe-N coordination content and favorable electronic structure.

### 3.2. Electrochemical Properties of the Catalysts

**Figure 6:**
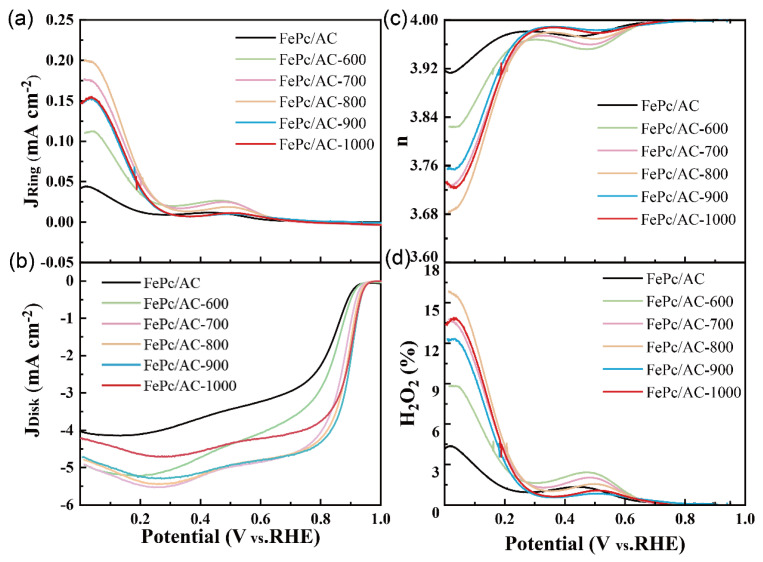
LSV curves showing the (**a**) ring and (**b**) disk current densities, (**c**) electron transfer number (n), and (**d**) H_2_O_2_ yield (%) for FePc/AC, FePc/AC-600, FePc/AC-700, FePc/AC-800, FePc/AC-900, and FePc/AC-1000 at 1600 rpm in a 0.1 M KOH solution at a scan rate of 10 mV s^−1^.

To investigate the differences in the ORR performances of the FePc/AC catalysts prepared using AC samples pyrolyzed at different temperatures, RRDE tests were conducted on them (FePc/AC, FePc/AC-600, FePc/AC-700, FePc/AC-800, FePc/AC-900, and FePc/AC-1000) in a 0.1 M KOH solution. The ring and disk current LSV curves of the samples are shown in Figure 6a,b, and their ORR performance parameters are summarized in Table 2. As shown in Table 2, the *E*_on_ of FePc/AC, FePc/AC-600, FePc/AC-700, FePc/AC-800, FePc/AC-900, and FePc/AC-1000 were 0.92, 0.93, 0.94, 0.95, 0.95, and 0.95 V vs. RHE, respectively. Further, the *E*_1/2_ of FePc/AC-600, FePc/AC-700, FePc/AC-800, FePc/AC-900, and FePc/AC-1000 were 0.83, 0.87, 0.88, 0.89, and 0.89 V vs. RHE, respectively. The more positive the *E*_1/2_ is, the smaller the overpotential of the reaction is, and the higher the ORR activity of the sample is [52]. The above results indicate that the *E*_on_ and *E*_1/2_ of the catalyst gradually shift to more positive values with increasing heat-treatment temperature of the carbon support, indicating that the ORR activity of the sample increases with increasing heating temperature. However, when the heat-treatment temperature reached 1000 °C, the *E*_1/2_ of FePc/AC-1000 remained essentially unchanged, and the ORR activity of the sample increased only slightly. The *J*_L_ of FePc/AC-600, FePc/AC-700, FePc/AC-800, FePc/AC-900, and FePc/AC-1000 were 3.67, 4.72, 5.23, 5.20, 5.13, and 4.56 mA cm^−2^, respectively. Notably, FePc/AC-1000 exhibited a significantly lower *J*_L_ than FePc/AC-900. This result is attributable to the lower porosity of FePc/AC-1000, which restricts O_2_ transport within the catalyst, leading to a decrease in *J*_L_.

Figure 6c,d show the n and H_2_O_2_% of all catalyst samples. As shown in Table 2, FePc/AC, FePc/AC-600, FePc/AC-700, FePc/AC-800, FePc/AC-900, and FePc/AC-1000 exhibit H_2_O_2_ production yields of 1.25, 1.72, 1.51, 1.22, 0.73, and 0.75%, respectively, at 0.4 V, and the average electron transfer numbers (n) are 3.972, 3.958, 3.965, 3.975, 3.991, and 3.988, respectively. These results indicate that all samples produce relatively low amounts of H_2_O_2_ during the ORR reaction, with the reaction primarily occurring via the four-electron pathway. Among the samples, FePc/AC-900 exhibited the lowest H_2_O_2_ production rate and the highest number of transferred electrons, which conferred it with a higher ORR activity. In Figure S1, we plotted the LSV curves of Pt/C and FePc/AC-900 to compare their performances.

**Figure 7:**
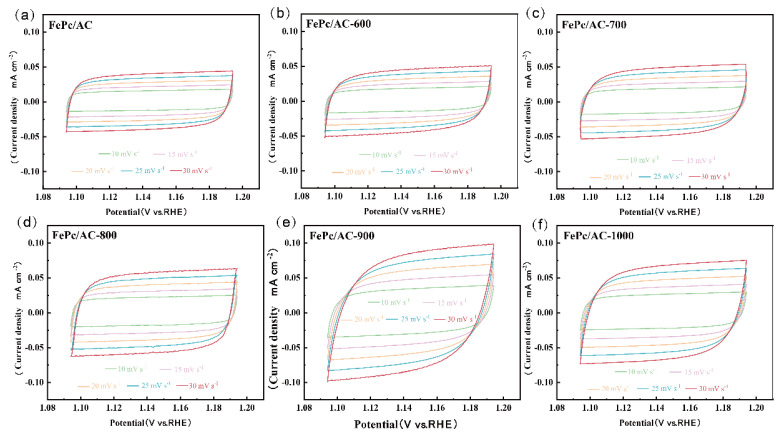
CV curves of the FePc catalysts in the non-Faradaic potential region (1.09–1.19 V vs. RHE): (**a**) FePc/AC, (**b**) FePc/AC-600, (**c**) FePc/AC-700, (**d**) FePc/AC-800, (**e**) FePc/AC-900, and (**f**) FePc/AC-1000. The scanning rate was varied to 10, 15, 20, 25, and 30 mV s^−1^.

CV measurements were conducted on the FePc/AC to FePc/AC-1000 catalysts in the non-Faradaic potential range (1.09–1.19 V vs. RHE) at varying scan rates (10, 15, 20, 25, and 30 mV s^−1^) to determine their double-layer capacitance (*C*_dl_). As shown in Figure 7a–f, the measured current densities of all six catalysts demonstrated a strictly linear relationship with the scan rate. The derived *C*_dl_ values of the FePc/AC, FePc/AC-600, FePc/AC-700, FePc/AC-800, FePc/AC-900, and FePc/AC-1000 samples were 1.29, 1.44, 1.56, 1.87, 2.59, and 2.15 mF cm^−2^, respectively.

**Figure 8:**
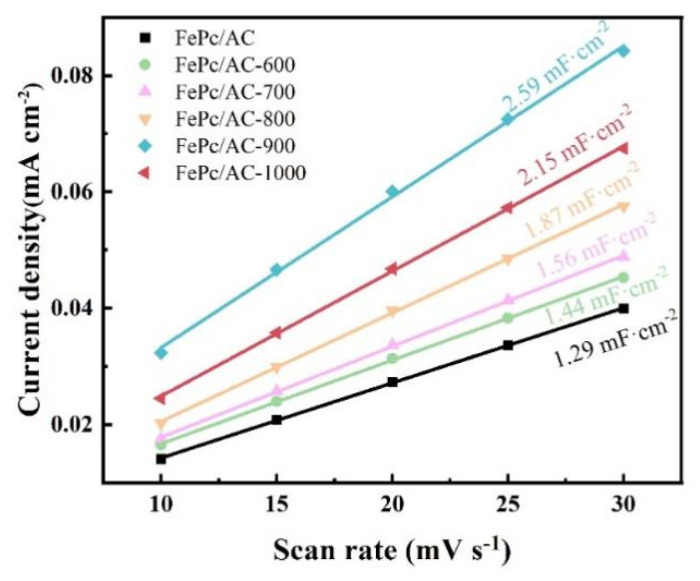
Double-layer capacitance (*C*_dl_) values of the FePc/AC to FePc/AC-1000 catalysts determined based on the scanning rate at a potential of 1.145 V vs. RHE, and the slope of the corresponding current–voltage curve.

The electrochemically active surface areas (ECSAs) of the FePc/AC, FePc/AC-600, FePc/AC-700, FePc/AC-800, FePc/AC-900, and FePc/AC-1000 catalyst samples were calculated using the relationship of ECSA = C_dl_/C_s_, where C_s_ was set to 40 μF/cm^2^ [53]. The value of C_dl_ is shown in Figure 8, the derived ECSA values were 32.25, 36.00, 39.00, 46.75, 64.75, and 53.75 cm^2^ for FePc/AC, FePc/AC-600, FePc/AC-700, FePc/AC-800, FePc/AC-900, and FePc/AC-1000, respectively. Notably, FePc/AC-900 exhibited the highest ECSA, suggesting optimal pore accessibility for electrolyte infiltration under ideal conditions (i.e., assuming minimal pore blockage by the binder during electrode preparation). The enhanced ECSA of FePc/AC-900 implies the formation of a well-developed gas/liquid/solid three-phase interface on the electrode surface, which correlates with its superior ORR activity compared to that of the other catalysts. Figure S2 shows that the electrochemically active area of the catalyst remained basically unchanged after multiple CV cycles.

**Figure 9:**
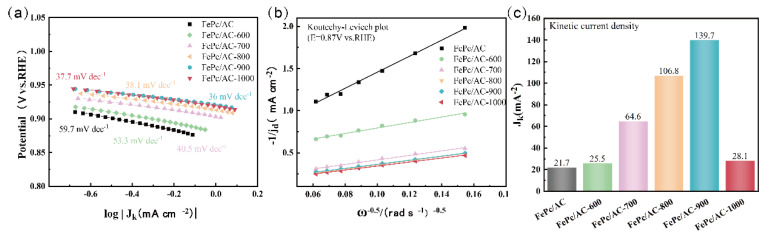
Electrochemical ORR performances of FePc/AC to FePc/AC-1000 catalysts in 0.1 mol KOH: (**a**) Tafel slope, (**b**) K–L slope, and (**c**) kinetic current density.

To systematically analyze the ORR kinetics of all catalyst samples, their Tafel slope, Koutecky–Levich (K–L) slope, and kinetic current density were investigated, and the results are presented in Figure 9. Figure 9a shows that the Tafel slope of FePc/AC-900 is 36 mV dec^−1^, significantly lower than those of FePc/AC (59.7 mV dec^−1^), FePc/AC-600 (53.3 mV dec^−1^), FePc/AC-700 (40.5 mV dec^−1^), FePc/AC-800 (38.1 mV dec^−1^), and FePc/AC-1000 (37.7 mV dec^−1^). Furthermore, the trend of the Tafel slope change aligns with the *E*_1/2_ variational trend of all catalyst samples, further confirming that FePc/AC-900 exhibits a superior ORR catalytic activity across a broader potential range. Additionally, RDE tests were conducted on FePc/AC-600 to FePc/AC-1000 samples at electrode rotational speeds ranging from 400 to 2500 rpm, and the K–L slope and kinetic current density (*J*_K_) of all samples were calculated. Figure 9b presents the K–L slopes of FePc/AC to FePc/AC-1000 samples at 0.87 V vs. RHE, revealing that the FePc/AC-600 to FePc/AC-1000 samples, which were pyrolyzed at high temperatures, exhibit smaller K–L slopes than the non-pyrolyzed FePc/AC, indicating that high-temperature pyrolysis enhances the ORR kinetics of the FePc/AC catalyst. Notably, FePc/AC-900 and FePc/AC-1000 possess similar K–L slopes and demonstrate faster ORR dynamics. Figure 9c presents the kinetic current densities of FePc/AC to FePc/AC-1000. The *J*_K_ values of FePc/AC-600 to FePc/AC-1000 are 21.7, 25.5, 64.6, 106.8, 139.7, and 28.1 mA cm^−2^, respectively, with FePc/AC-900 exhibiting the highest *J*_K_ value. These findings suggest that FePc/AC-900 demonstrates the most remarkable ORR kinetic performance owing to its rich pore structure, large defect density, and maximum content of Fe-N active sites.

We compared our FePc/AC-900 with cutting-edge M-N-C catalysts. Table 3 summarizes the electrochemical intrinsic activity, where FePc/AC-900 exhibits an E_1/2_ (0.89 V vs. RHE) that surpasses Fe/NC-NaCl (0.832 V vs. RHE). This comparison not only validates the superior ORR activity of FePc/AC-900 but also underscores the innovation of our carbon carrier microstructure regulation strategy—achieving higher activity without relying on noble metals.

## 4. Conclusions

In this study, AC was employed as a carbon precursor to systematically investigate the influence of the carbon microstructure on the ORR performance of Fe-N-C catalysts by precisely controlling the carbon pyrolysis temperature (600–1000 °C). The findings revealed that the heat-treatment temperature critically governs the catalytic activity of the loaded non-metal catalyst by affecting the conductivity, defect density, and specific surface area of the carbon support. As the pyrolysis temperature of AC increased from 600 to 900 °C, the resultant increase in the conductivity and defect density of the carbon carrier synergistically improved the ORR activity of the Fe-N-C catalysts. However, increasing the pyrolysis temperature further to 1000 °C induced structural collapse, leading to a reduction in the specific surface area and pore volume, which adversely affected the ORR performance. The FePc/AC-900 catalyst, derived from AC pyrolyzed at 900 °C, exhibited optimal properties—including high conductivity, maximized defect density, and a well-developed pore structure—facilitating exceptional FePc loading and abundant Fe-N active sites. Consequently, it exhibited an outstanding ORR activity, with an *E*_1/2_ of 0.89 V and *E*_on_ of 0.95 V vs. RHE.

This study demonstrates a nonlinear relationship between the pyrolysis temperature of carbon and the catalytic performance of the loaded catalyst, revealing that an optimal pyrolysis temperature (900 °C) provides balanced structural and electronic properties. This work provides critical insights for designing high-performance M-N-C catalysts by rationally modulating the microstructure of carbon supports, paving the way for advanced ORR electrocatalysts.

## Figures and Tables

**Table 1:** Electrical conductivities of the AC samples pyrolyzed at different temperatures.

		1 MPa	2 MPa	3 MPa
	AC	8.87 × 10^−4^	1.36 × 10^−3^	1.62 × 10^−3^
Electrical conductivity S/m	AC-600	1.87 × 10^−2^	2.62 × 10^−2^	3.01 × 10^−2^
	AC-700	2.64 × 10	3.47 × 10^2^	4.22 × 10^2^
	AC-800	5.59 × 10^3^	8.23 × 10^3^	9.84 × 10^3^
	AC-900	1.52 × 10^4^	2.37 × 10^4^	2.86 × 10^4^
	AC-1000	2.44 × 10^4^	3.28 × 10^4^	3.68 × 10^4^

**Table 2:** Summary of the ORR performances of FePc/AC, FePc/AC-600, FePc/AC-700, FePc/AC-800, FePc/AC-900, and FePc/AC-1000 catalysts.

Sample	*E*_on_ (V vs. RHE)	*E*_1/2_ (V vs. RHE)	*J*_L_ at 0.4 V (mA cm^−2^)	H_2_O_2_% at 0.4 V (%)	*n* at 0.4 V
FePc/AC	0.92	0.81	3.67	1.25	3.972
FePc/AC-600	0.93	0.83	4.72	1.72	3.958
FePc/AC-700	0.94	0.87	5.23	1.51	3.965
FePc/AC-800	0.95	0.88	5.20	1.22	3.975
FePc/AC-900	0.95	0.89	5.13	0.73	3.991
FePc/AC-1000	0.95	0.89	4.56	0.75	3.988

**Table 3:** Electrochemical intrinsic activity of representative M-N-C type catalysts in recent years.

Catalyst	E_1/2_ (V vs. RHE)	J_l_ (mA cm^−2^)	Tafel (mv dev^−1^)
Fe/NC-NaCl [54]	0.83	5.20	66.3
Zn-N-C [55]	0.87	5.50	60.0
FePc/AC-900	0.89	5.13	36.0
FePc_0.5_/PBC [13]	0.91	5.03	34.4
Fe_0.1_CNT@NHC [14]	0.92	6.08	65.1

## Data Availability

The raw data supporting the conclusions of this article will be made available by the authors on request.

## Supplementary Materials

The following supporting information can be downloaded at: https://www.mdpi.com/article/10.3390/nano15171327/s1, Figure S1: LSV curves Pt/C and FePc/AC-900 at 1600 rpm in a 0.1 M KOH solution at a scan rate of 10 mV s^−1^; Figure S2: Cyclic voltammograms of FePc/AC-900 catalyst at different cycles. Cycling at 500 mV/s from 0–1.23 V vs.RHE in 0.1 M KOH.
