# Supplementary material for: Effect of the Microstructure of Carbon Supports on the Oxygen Reduction Properties of the Loaded Non-Noble Metal Catalysts
Source: Nanomaterials (Basel). 2025 Aug 29;15(17):1327. doi: 10.3390/nano15171327 (PMC12430596; doi:10.3390/nano15171327)
Supplement: Supplementary file 1 [file nanomaterials-15-01327-s001.zip › nanomaterials-3744640-supplementary.pdf]

Supplementary Materials

Effect of the Microstructure of Carbon Supports on the Oxygen Reduction Properties of the Loaded Non-Noble Metal Catalysts

Dan Ma[1], Yudong Zhang[1,2,*], Menghan Liang[1], Runyu Niu[1], Yao Ge[2], Yanan Zou[3], Xiaorui Dong[1,*]

1 School of Energy and Power Engineering, North University of China, Taiyuan, 030051, China
2 Chongqing University Industrial Technology Research Institute, Chongqing University, Chongqing 400030, China
3 College of Mechanical Engineering, University of South China, Hengyang 421001, PR China
* Correspondence: Correspondence: 20230020@nuc.edu.cn;

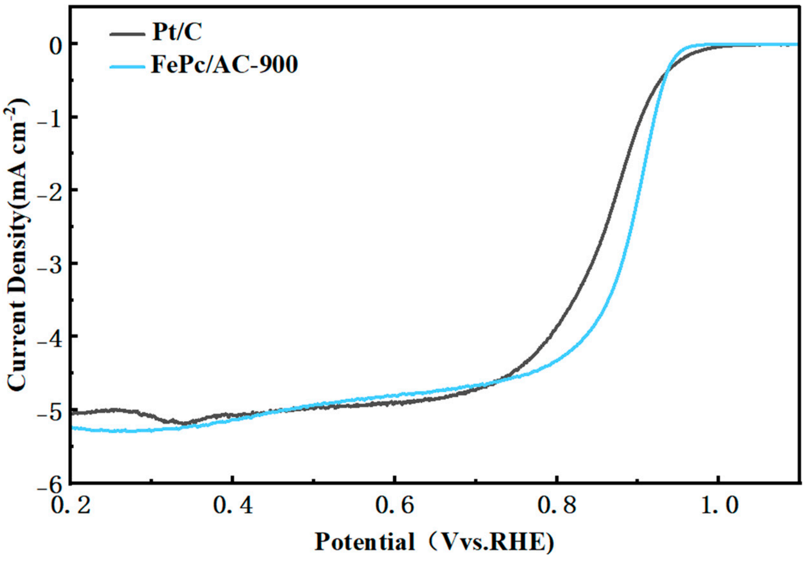

Figure S1. LSV curves Pt/C and FePc/AC-900 at 1600 rpm in a 0.1 M KOH solution at a scan rate of 10 mV $^{s-1}$.

Figure S1. Shows the LSV curves of the FePc/AC-900 catalyst we prepared and the commercial Pt/C catalyst under the same test conditions. The results show that the initial potentials of Pt/C and FePc/AC-900 are 0.97 and 0.95 V vs. RHE, respectively, and the half-wave potentials are 0.86 and 0.89V vs. RHE, respectively. This result indicates that the catalyst FePc/AC-900 prepared in this paper has better ORR catalytic activity, suggesting that it has the potential to serve as an alternative to commercial Pt/C catalysts in the ORR field.

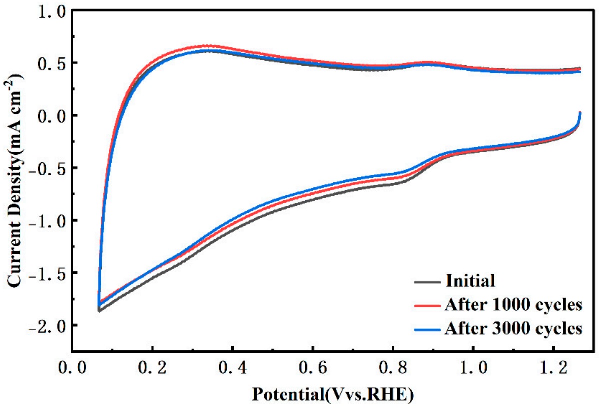

Figure S2. Cyclic voltammograms of FePc/AC-900 catalyst at different cycles. Cycling at 500 mV/s from 0-1.23Vvs.RHE in 0.1 M KOH.

Figure S2 shows that after scanning the sample for 1000 and 3000 cycles within the non-Faraday range of 1.0–1.23V vs. RHE, the CV curve changed little, indicating that the ECSA of the catalyst remained basically unchanged and proving that the electrochemically active area of the catalyst remained basically unchanged after multiple CV cycles.
